# Recent progress on methods for estimating and updating large phylogenies

**DOI:** 10.1098/rstb.2021.0244

**Published:** 2022-10-10

**Authors:** Paul Zaharias, Tandy Warnow

**Affiliations:** Department of Computer Science, University of Illinois Urbana-Champaign, Urbana, IL 61801, USA

**Keywords:** phylogeny estimation, multiple sequence alignment, phylogenetic placement, phylogenomics, taxon identification, maximum likelihood

## Abstract

With the increased availability of sequence data and even of fully sequenced and assembled genomes, phylogeny estimation of very large trees (even of hundreds of thousands of sequences) is now a goal for some biologists. Yet, the construction of these phylogenies is a complex pipeline presenting analytical and computational challenges, especially when the number of sequences is very large. In the past few years, new methods have been developed that aim to enable highly accurate phylogeny estimations on these large datasets, including divide-and-conquer techniques for multiple sequence alignment and/or tree estimation, methods that can estimate species trees from multi-locus datasets while addressing heterogeneity due to biological processes (e.g. incomplete lineage sorting and gene duplication and loss), and methods to add sequences into large gene trees or species trees. Here we present some of these recent advances and discuss opportunities for future improvements.

This article is part of a discussion meeting issue ‘Genomic population structures of microbial pathogens’.

## 1. Introduction

Large-scale phylogeny estimation presents substantial computational and statistical challenges: the most accurate methods are often likelihood-based methods (maximum likelihood or Bayesian inference) that can use substantial time and memory to produce reliable trees. Multiple sequence alignment (a precursor to phylogeny estimation) is also challenging, especially on large datasets that have high rates of evolution. Furthermore, species tree estimation presents additional challenges due to heterogeneity in phylogenetic trees between different loci, which can result from processes such as incomplete lineage sorting (ILS), gene duplication and loss (GDL) and horizontal gene transfer (HGT) [1]. Yet because dense taxonomic sampling has been seen to improve phylogenetic accuracy [2], the interest in statistically rigorous methods for large-scale phylogeny estimation (whether of gene trees or species trees) has not abated.

The past decade has produced methods for alignment and phylogeny estimation that have excellent accuracy on small- to moderate-sized datasets, but only a few of these methods can analyse even moderately large datasets (1000 sequences). Some of the phylogeny estimation methods with the best scalability are distance-based (e.g. FastME [3]). However, several studies (e.g. [4]) have shown that maximum-likelihood (ML) methods tend to be more accurate than distance-based approaches on large datasets, especially under high rates of evolution.

Because ML phylogeny estimation can be computationally intensive (both for time and memory), substantial effort has been made to improve the running time through careful implementation of the numerical calculations and use of parallelism (see recent surveys in [5–7]). Despite the advances in the past decade, the construction of very large ML phylogenies (e.g. gene phylogenies of 100 000 or more sequences or 10 000 whole genomes) is very difficult using standard approaches, except perhaps when supercomputers are available.

Divide-and-conquer is a natural technique to speed up computationally intensive analyses: for example, rather than estimating a tree on a dataset with 100 000 sequences, the input could be divided into many smaller datasets (perhaps 100 datasets with approximately 1000 sequences each), trees could be estimated on each subset, and then combined into a tree on the entire dataset. An obvious divide-and-conquer technique would use taxonomic information to define the subsets; however, using taxonomies presents potentially significant challenges. For example, when estimating gene trees, discordance between gene trees and species trees (resulting from various biological processes) can mean that taxonomically derived decompositions do not form connected subtrees in the true gene trees. An additional complication that impacts all estimation problems is that taxonomies can have mistakes; as a result, techniques that use taxonomic information are often combined with opportunities for the user to correct potential mistakes. Finally, taxonomies may not include all the sequences in the input. Despite the challenges in using taxonomies, they can be very useful in constraining the search space, and so result in reduced running time. PyPHLAWD [8] and PhyLoTA [9] are two such techniques, and strategies like these have been used in phylogenomic analyses (e.g. [10,11]).

In this paper, we present new divide-and-conquer techniques to scale computationally intensive but highly accurate methods to large and even ultra-large datasets, without using taxonomic information. We show how divide-and-conquer can improve many steps in a phylogenomic pipeline, starting with large-scale multiple sequence alignment (a precursor to phylogeny estimation) and ending with updating large trees. However, these are not the only recently developed divide-and-conquer methods; this issue also has a paper by Achtman *et al.* [12] that presents another divide-and-conquer method and uses it to construct a very large bacterial tree. Thus, divide-and-conquer is a powerful technique that can be used in different ways for large-scale phylogeny and alignment estimation.

## 2. Recent advances in multiple sequence alignment

Multiple sequence alignment (MSA) is a precursor to phylogeny estimation as well as to other bioinformatics problems, such as sequence classification and protein function prediction. When the input is a set of sequences for a group of closely related individuals, then techniques that operate by inferring pairwise alignments to a single reference sequence can have good accuracy; however, the estimation of multiple sequence alignments for more distantly related sequences requires other techniques. There are many well-established methods (surveyed in [13]), but only some of these provide good accuracy on large sequence datasets, especially when they have evolved under high rates of evolution.

Divide-and-conquer techniques have proved very powerful tools in scaling the most accurate alignment methods to large datasets. These methods (e.g. [14–18]) divide the input sequence dataset into disjoint subsets, produce alignments on each subset using a selected ‘base method’ and then merge the subset alignments together. When combined with iteration (so that each iteration uses the previous iteration’s alignment to compute a new tree and then decomposes the dataset using the tree), the methods can produce highly accurate alignments and trees, typically in just a few iterations. PASTA [16] is one of the most accurate and scalable divide-and-conquer methods for co-estimating alignments and trees. MAFFT [19] is the default method for subset alignment for the PASTA pipeline, but other methods can also be used. For example, Nute & Warnow [20] used BAli-Phy [21], a Bayesian alignment method, as the subset aligner and found that this modified PASTA pipeline improved accuracy compared to default PASTA.

**Figure 1.**
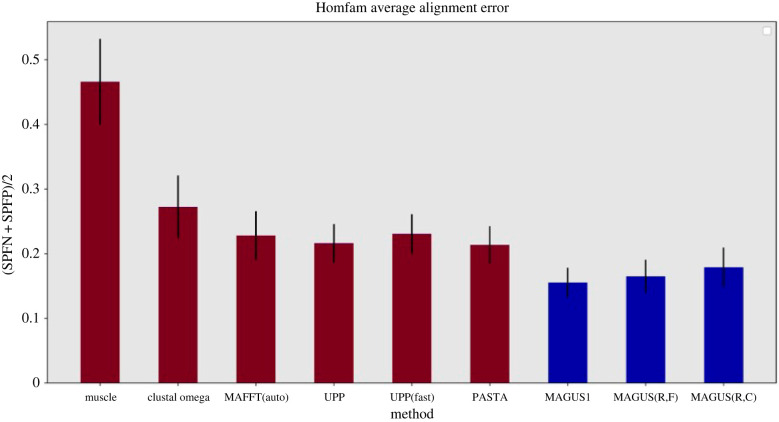
Average alignment error on 19 datasets with 10 099–93 681 sequences. The datasets are from the Homfam [24] collection of benchmark protein datasets with alignments defined by secondary and tertiary protein structures. Alignment error is based on pairwise homology statements for each alignment, where two letters that are in the same column of an alignment are considered homologous according to that alignment. The fraction of the pairwise homologies (defined by the reference alignment) that are not in the estimated alignment is the sum-of-pairs false negative (SPFN) error rate, and the fraction of the pairwise homologies in the estimated alignment that are not in the reference alignment is the sum-of-pairs false positives (SPFP) error rate. Results are averaged over the datasets where all methods completed (Muscle segfaulted on two). Error bars show standard error. Reproduced from Smirnov [18] under the Creative Commons Attribution License.

A new and promising divide-and-conquer strategy is used in MAGUS [17,18], a recently developed MSA method that is closely related to PASTA. Specifically, whereas PASTA merges a set of disjoint alignments by merging selected pairs of alignments and then using transitivity to complete the merger, MAGUS achieves the merger by first computing a graph where the vertices represent the sites in the alignments, and then clustering the sites together to define the merged alignment. This clustering step, performed using the Graph Clustering Merger (described in [17]), is the key to the improved accuracy that MAGUS has over PASTA, as all other algorithmic differences between MAGUS and PASTA are very minor. As demonstrated in [22], the 'graph clustering merger' is an effective strategy for solving the maximum weight trace problem [23] in the context of merging alignments. Figure 1 provides a sample of results from Smirnov [18], which show that MAGUS and its recursive version are more accurate than leading alignment methods on large biological benchmark datasets and simulated datasets (up to 1 000 000 sequences).

## 3. Recent advances in maximum-likelihood tree estimation

ML gene tree estimation is one of the core problems in phylogeny estimation. One of the reasons for its popularity is that ML tree estimation has been proven to be a statistically consistent estimator of the phylogeny under standard sequence evolution models, which means that as the sequence length increases the method will converge to the true tree with probability increasing to 1 [25]. However, finding the optimal ML tree is NP-hard [26] and so the best heuristics, such as RAxML [27] and IQ-TREE [28], use many different strategies to search for the tree optimizing the likelihood score. FastTree 2 [29] is a very fast heuristic that does not make a very substantial attempt to optimize likelihood (and hence does not find very good ML scores).

RAxML has been modified over the years to improve scalability to large datasets, and the current version, RAxML-NG [30], is able to analyse very large datasets. However, a recent study [31] showed that RAxML-NG, using 16 CPUs, did not converge on a 10 000-sequence dataset even after a week. By contrast, the 2010 paper introducing FastTree 2 [29] showed it was able to estimate an ML tree with 237 882 distinct sequences in 22 h, and a recent study [18] demonstrated that FastTree 2 was able to produce a tree on 1 000 000 sequences in approximately 5 days using 32 CPUs. Thus, FastTree 2 clearly dominates RAxML for speed.

Interestingly, the accuracy comparison between RAxML and FastTree 2 has mixed results. A 2011 study showed the two had very similar topological accuracy [32], but later studies have shown that FastTree 2 can be less accurate than RAxML when the input alignment contains many fragmentary sequences [31,33] or is otherwise very gappy [34]. In addition, a recent study showed reduced accuracy for FastTree 2 when the sequences have evolved under heterotachy [31]. By contrast, RAxML and to a somewhat lesser extent also IQ-TREE 2 [35] seem more robust to those conditions [31].

Several strategies have been developed to overcome the burden of computationally intensive ML analyses. Some of these (e.g. DACTAL [36]) operate by dividing the input set into overlapping subsets, constructing trees on the subsets, and then using supertree methods to merge the subset trees into a tree on the full dataset. This is a natural approach to large-scale tree estimation [37], but the choice of decomposition strategy can impact the final accuracy, and random decompositions in particular can produce poor supertrees [38]. Furthermore, the requirement to use supertree methods (which are not yet very fast) constrains the scalability of these approaches [39].

**Figure 2.**
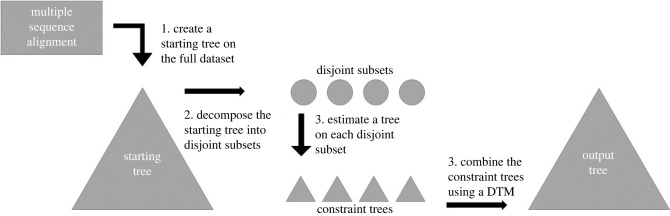
DTM pipeline for constructing a tree from an input sequence alignment using ML. (1) A starting tree is computed (e.g. using FastTree 2 or IQ-TREE 2 [35]). (2) Edges are deleted from the starting tree to produce small subsets. (3) Trees are estimated on the subsets using a selected ML method (e.g. IQ-TREE 2 or RAxML-NG). (4) The selected 'disjoint tree mergers' (DTM) method merges the disjoint trees into a tree on the full dataset. DTM pipelines that operate from multi-locus inputs and compute species trees have also been developed, with suitable adjustments to the algorithmic steps. Reproduced from Park *et al*. [31] under the Creative Commons Attribution License.

To overcome these limitations, a new type of divide-and-conquer approach, disjoint tree merging (DTM), has been developed. In this approach (figure 2), an initial tree is computed on the input. Then edges are deleted from the tree until each subset is small enough (below a user-provided threshold). Then trees are estimated on each subset, and finally merged into a tree on the full dataset. This four-stage approach divides the input dataset into disjoint rather than overlapping sets, and hence requires additional information, such as a distance matrix or a guide tree, in order to merge the subset trees into a full tree.

Methods that can merge a set of leaf-disjoint trees into a single tree are called ‘disjoint tree mergers’ (DTMs), and pipelines that use DTMs can be used to estimate both gene trees and species trees. Several DTMs have been developed, starting with NJMerge [40], TreeMerge [41], Constrained-INC [42,43] and most recently including the guide tree merger (GTM) [44]. Of these, the GTM has been shown to be very fast and generally as accurate as the previously developed DTMs. When the initial tree and the subset trees are all estimated using statistically consistent methods, then DTM pipelines using GTM (as well as the other DTM methods listed above) are provably statistically consistent.

**Figure 3.**
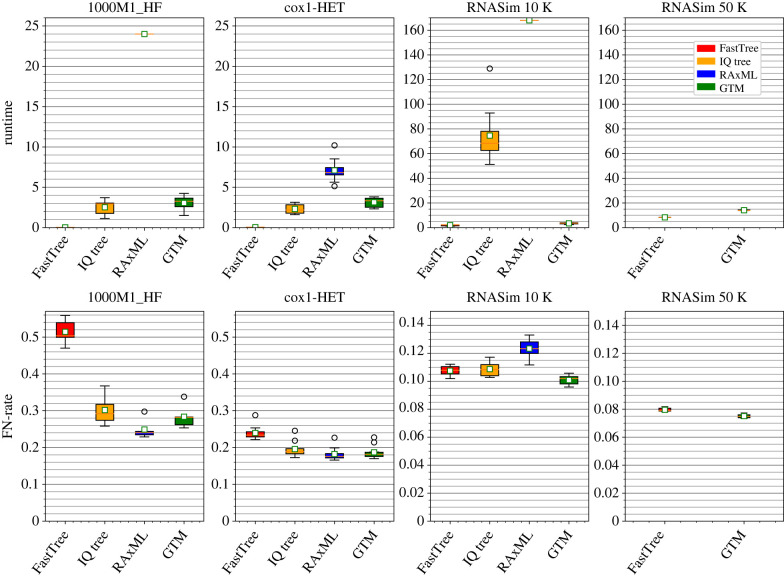
Comparison of standard ML methods (RAxML-NG, IQ-TREE 2 and FastTree 2) to a divide-and-conquer pipeline using the guide tree merger (GTM) on four simulated datasets with 1000–50 000 sequences. 1000M1-HF datasets each have 1000 sequences that evolved under a GTRGAMMA+indel model and include fragmentary sequences, Cox1-HET datasets each have 2341 sequences that evolved with heterotachy, and the RNASim [16] datasets have 10 000–50 000 sequences each and evolved under selective pressures to maintain the RNA secondary structure. Top: running time (hours), bottom: missing branch (FN) error rates across 10 replicates per model condition. Results not shown for IQ-TREE 2 and RAxML on the RNASim 50K dataset because IQ-TREE 2 failed to return a tree within the allowed time (24 h for the two smaller datasets and 168 h for the two larger datasets) and RAxML-NG produced trees with at least 99.96% FN error. Adapted from [31] under the Creative Commons Attribution License. (Online version in colour.)

Figure 3 shows results from [31] comparing a DTM pipeline using GTM to two leading ML methods (RAxML-NG and IQ-TREE 2). For topological error, we report the false negative error rate, which indicates the proportion of the non-trivial splits in the true tree that are not produced in the estimated tree. The GTM pipeline matches or improves on the topological accuracy of both IQ-TREE 2 and FastTree 2 and is competitive with RAxML-NG, while being much faster than RAxML-NG. A comparison on the largest dataset with 50 000 sequences, limited to 168 h (one week) of analysis, shows that only the GTM pipeline and FastTree 2 are acceptable: RAxML-NG has nearly 100% false negative error on that model condition while IQ-TREE 2 fails to return a tree at all due to memory issues.

To understand this performance, we note that [31] used RAxML-NG in default mode (10 random starting trees and 10 random sequence addition parsimony trees). The poor tree accuracy is consistent with RAxML-NG completing only a few rounds of heuristic search and so returning a tree that is close to the starting tree. It is possible that RAxML-NG might have been able to produce a good tree on this dataset using a different starting tree (e.g. using FastTree 2). Thus, while [31] does show advantages to using a GTM-pipeline for large-scale ML compared to both IQ-TREE 2 and RAxML-NG, future work is needed to better understand how to use RAxML-NG and IQ-TREE 2 to estimate ultra-large trees without requiring very large amounts of memory or time.

## 4. Recent advances in species tree estimation

A traditional approach to multi-locus species tree estimation concatenates the individual gene sequence alignments into a ‘supermatrix’ and estimates a tree on the supermatrix, often using ML. These ‘concatenation analyses’ are appealing but can be very computationally expensive: the ML analysis of the 48 bird genomes in [45] took 250 CPU years, and the ML concatenation pipeline of [46] took approximately 33 000 CPU hours (about 3.8 CPU years) to build a tree on 10 575 genomes. In addition, because different genomic regions can have different evolutionary histories due to processes such as ILS and GDL, the use of concatenation (which assumes that all the sites evolve down a single tree topology) has been significantly criticized [47,48]. As a result, new approaches based on statistical models for gene evolution within species trees have been developed and are now increasingly used, and some of these approaches are very scalable. Here we present recent advances for species tree estimation that provide high accuracy and scalability.

### (a) Species tree estimation in the presence of incomplete lineage sorting

The problem of species tree estimation in the presence of ILS is very well studied. Although species trees have traditionally been estimated using ML and other methods on a concatenation of the individual gene sequence alignments, this approach has been shown to be statistically inconsistent when there is gene tree heterogeneity due to ILS [49].

One of the statistically consistent approaches for species tree estimation when ILS is present operates by estimating gene trees for each gene and then combining the gene trees. These ‘summary methods’ are generally faster than concatenation (especially on large datasets). Two of the best-known methods are MP-EST [50] and ASTRAL [51], but ASTRAL is generally faster on large datasets. ASTRID [52] and DISTIQUE [53] are two other fast and scalable summary methods that are often comparable in accuracy to ASTRAL [53], but ASTRAL is more frequently used than ASTRID. All summary methods are impacted by gene tree estimation error (a common occurrence when gene sequence alignments are short or otherwise have low ‘phylogenetic signal’), and under conditions where all gene trees have low accuracy, concatenation analyses can be more accurate than even the best summary methods [54].

Alternative approaches have been developed that avoid these problems and that also provide statistical guarantees in the presence of ILS. One such example is SVDquartets [55], a method that uses properties of the multi-species coalescent model to estimate quartet trees and then combines the quartet trees into a tree on the full set of species. SVDquartets (and its variants, e.g. SVDquest [56]) can provide superior accuracy compared to summary methods under conditions with high gene tree estimation error [54], but more study is needed to understand the empirical conditions under which they are more reliable than standard ML concatenation analyses. Finally, co-estimation of gene trees and species trees is also more robust to conditions where gene sequence alignments have low phylogenetic signal, and Bayesian co-estimation methods such as StarBEAST2 [57] can provide outstanding accuracy. However, current Bayesian co-estimation methods are limited to small numbers of species and loci due to computational requirements (though see [58]).

For these reasons, summary methods such as ASTRAL have become a mainstream approach to species tree estimation on datasets with large numbers of species. ASTRAL constructs an unrooted species tree from a set of unrooted gene trees by solving the ‘maximum quartet support supertree’ problem (i.e. finding a species tree that agrees with as many quartet trees induced by the input gene trees as possible). Since this is an NP-hard problem, the default setting for ASTRAL solves the problem within a constrained search space that is computed from the input gene trees. Specifically, ASTRAL only considers those candidate species trees that draw their bipartitions from a constraint set that contains the input gene tree bipartitions and potentially some additional bipartitions. Although ASTRAL runs in polynomial time, its worst-case runtime is nearly quadratic in the number of distinct bipartitions found in the constraint set. Since this constraint set can be quite large when there is substantial heterogeneity between gene trees and large numbers of genes, ASTRAL can sometimes take a long time to complete (i.e. days).

To reduce ASTRAL’s runtime and improve scalability to large datasets, two high-level techniques have been developed. The first is the use of DTM pipelines, described above in the context of gene tree estimation, but adapted to enable species tree estimation on multi-locus datasets. As shown in [41,44], DTM pipelines greatly reduce the running time for ASTRAL on large taxon sets and can also improve accuracy. The divide-and-conquer pipeline presented in [12] is also used to estimate a species tree, with ASTRAL the method for constructing species trees on each subset. Although the details of the pipeline in [12] are slightly different from the specific DTM pipeline structure given in figure 2, clearly the divide-and-conquer pipeline in [12] is a DTM pipeline for species tree estimation.

The second technique operates by replacing the constraint set that ASTRAL computes from the input with a smaller constraint set. One such approach uses ‘external constraints’, for example partial information about the species tree, in order to reduce the constraint set size. The ASTRAL codebase was recently enhanced by such a technique [59], and we refer to its usage as ‘ASTRAL-J’ to reflect the flag used in ASTRAL given external constraints. Another approach runs ASTRID on a collection of subsamples of the gene trees, so that each ASTRID analysis of each subsample produces a candidate species tree. The bipartitions from those estimated trees are then used as the constraint set for ASTRAL. This approach, called ‘FASTRAL’ [60], is provably statistically consistent under the multi-species coalescent model. Furthermore, FASTRAL is generally similar in accuracy to ASTRAL while being much faster when the number of species and/or genes is large enough [60]. Finally, FASTRAL-J, a combination of FASTRAL and ASTRAL-J, has been developed, which provides runtime advantages over ASTRAL-J and comparable accuracy [61].

### (b) Species tree estimation in the presence of gene duplication and loss

Genes can evolve with duplication and loss (GDL), in which case a given organism can have multiple copies of a given gene. When phylogenies are computed on datasets with more than one gene copy in a given species, the gene trees that are produced will have leaves for each of these copies. As a consequence, the phylogeny for that gene (called a ‘gene family tree’) can have multiple leaves corresponding to these copies, each labelled by the same species. These gene family trees are called ‘MUL-trees’ to distinguish them from single-copy trees [62].

Here we describe four techniques for estimating species trees from genes that evolve with GDL. The first is to eliminate those genes that evolve with GDL and restrict instead to those genes that are single-copy in every organism. This practice reduces available data, and so raises the concern that accuracy could be reduced. The second approach uses methods to detect orthology, so that the multi-copy gene family can be reduced to single-copy genes. However, orthology detection is still not reliably solved well [63], and so this approach also has some problems. The third approach co-estimates gene family trees and species trees from the sequence alignments. Phyldog [64] is the best known of these approaches, and uses a statistically rigorous approach. Although it is highly accurate, it is computationally intensive and limited to very small datasets.

The final approach constructs the species trees from the gene family trees and some methods using this approach have strong theoretical guarantees and can be very fast. For example, a recent theoretical advance is the proof that ASTRAL-multi [65] and ASTRAL-one [66], two modifications of ASTRAL to enable them to estimate species trees from MUL-trees, are statistically consistent under statistical models of gene evolution that allow for GDL [66,67]. However, these statistically consistent methods are not as accurate as ASTRAL-Pro [68], a variant of ASTRAL recently developed specifically to address GDL [68]. Other methods that can estimate species trees from a set of MUL-trees have been developed, with gene tree parsimony the most well known (e.g. DupTree [69]), but also including MixTrEm-DLRS [70], MulRF [71], FastMulRFS [72] and SpeciesRax [73]. While not all of them have been compared to ASTRAL-Pro, those that have been evaluated have not been shown to be as reliably accurate as ASTRAL-Pro [74].

**Figure 4.**
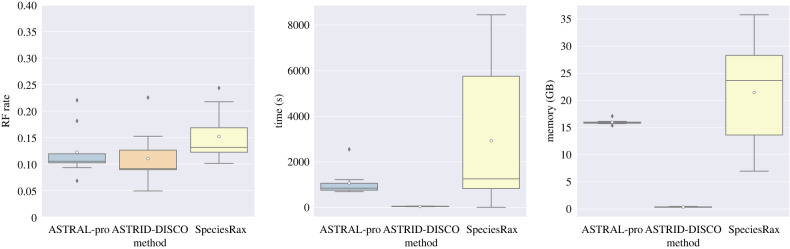
Species tree error (Robinson–Foulds (RF) error rates), wall clock running time (s) and peak memory usage of ASTRAL-Pro, ASTRID-DISCO and SpeciesRax on simulated datasets (evolved under GDL and ILS) of 1001 species and 50 estimated gene trees. All estimated and model trees are fully resolved, so the RF error rate is the fraction of bipartitions defined by internal edges of the model tree that are not in the estimated tree. Reproduced from [75] under the Creative Commons Attribution Non-Commercial License. (Online version in colour.)

Tree-decomposition provides another way of combining MUL-trees. In a tree-decomposition approach, each gene family tree is decomposed into a set of single-copy trees, and then the resultant set of single-copy trees is given to a selected summary method, such as ASTRAL or ASTRID. There are several such tree-decomposition methods, with DISCO [75] being a recent and promising technique. As seen in figure 4, using DISCO with ASTRID on a dataset with 1000 species produces a tree that is more accurate than ASTRAL-Pro and SpeciesRax, while being much faster and having lower memory requirements than both methods.

## 5. Recent advances in updating large trees

Once a large tree is estimated, if new sequence data become available, then starting all over is undesirable (especially since the first tree may have already required a great deal of computational effort and time). Hence, the problem of updating a tree by adding newly found sequences into the tree becomes relevant. The step of adding a sequence into a phylogeny is called ‘phylogenetic placement’ and it can be used both for gene trees and for species trees.

The methods described in this section are also relevant to understanding microbial diversity: given a sequence, placing it into a taxonomy makes it possible to characterize the sequence taxonomically, and so also enables an assessment of microbial diversity in a population [76–79]. This approach is particularly relevant for characterizing novel sequences (i.e. sequences that are not in public databases) and the accuracy of the taxonomic assignment improves on larger trees [79]. Therefore, methods for placing sequences into large trees also have utility for assessment of microbial diversity.

Phylogenetic placement is also useful when the input sequence dataset exhibits sequence length heterogeneity. For example, FastTree 2 can have poor topological accuracy on datasets with fragmentary sequences [33,34], with the consequence that in some conditions constructing trees on the full-length sequences and then using phylogenetic placement to add the remaining sequences can be more accurate than FastTree 2 on a good alignment [33].

### (a) Adding sequences to gene trees

One of the earliest methods for phylogenetic placement is pplacer [80]. The input is a binary tree with sequences at the leaves in an alignment, ML numeric parameters (e.g. branch lengths and substitution rate matrix) on the tree for that alignment, and a set of query sequences that need to be added into the tree. The approach used in pplacer is likelihood-based, with ML or Bayesian options both available; here we describe the ML version. Given query sequence *q*, pplacer seeks the edge in the tree where attaching *q* would optimize the ML score. Because pplacer is likelihood-based, this approach can be computationally intensive [81].

Other phylogenetic placement methods have been developed that seek to improve scalability to larger trees or reduce running time (e.g. UShER [82], RAPPAS [83], EPA-ng [84], APPLES [81] and APPLES-2 [85]). EPA-ng is likelihood-based and has been optimized for ‘batch processing’ of query sequences, so that the cost of performing phylogenetic placement of a large number of query sequences is much less than the cost of placing them one-by-one. EPA-ng has slightly reduced accuracy compared to pplacer. APPLES is a very fast distance-based method that places each query sequence into the tree so as to minimize the weighted least-squares error. APPLES-2 is an improvement on APPLES with respect to accuracy and running time, and also scales to at least 200 000 sequences. Recent studies [81,85,86] show that APPLES and APPLES-2 can run on trees with 200 000 leaves and are much faster than both pplacer and EPA-ng; however, even APPLES-2 does not match the accuracy of pplacer. UShER is parsimony-based and very fast, but has not been compared to pplacer, APPLES, or APPLES-2, while RAPPAS, which is based on k-mers, is very fast but not as accurate as EPA-ng or pplacer [83]). Thus, the highest accuracy in phylogenetic placement is obtained using likelihood-based methods, but these tend to be relatively computationally intensive compared to other approaches, especially distance-based or k-mer-based methods.

Recently, two divide-and-conquer methods, pplacer-SCAMPP [86] and pplacer-DC (pplacer-Divide-and-Conquer) [87], were developed in order to improve accuracy for phylogenetic placement when inserting into trees that are too large for pplacer. Here we describe the pplacer-SCAMPP approach, as a comparison of pplacer-SCAMPP with pplacer-DC on the RNASim VS datasets reported in [86,87] shows that pplacer-SCAMPP is faster, uses less memory, and is more accurate than pplacerDC. In addition, pplacer-SCAMPP is able to scale to trees with 200 000 leaves, whereas pplacer-DC scales only to 100 000 sequences [86,87].

The pplacer-SCAMPP pipeline uses four stages to insert a query sequence *q* into a tree *T*. First, a leaf that has the greatest sequence similarity to *q* is found. In the second stage, a contiguous subtree *t* is extracted from *T* that includes the nearest leaf and up to *N* − 1 additional leaves (where *N* = 2000 when the SCAMPP framework is used with pplacer). In the third stage, pplacer is used to insert the query sequence into the subtree *t* (i.e. an edge *e* in the subtree *t* is identified); since *N* was set to be only 2000, pplacer can complete on this dataset. Finally, in the fourth stage, an edge *e*′ in the tree *T* is found corresponding to the edge *e*, and the query sequence is placed into edge *e*′. By design, this four-stage approach can be modified to suit a different phylogenetic placement method, so that methods that can run on larger trees can have larger values for *N*. For example, when using the SCAMPP framework with EPA-ng, *N* is set to 10 000. Every stage of this pipeline, other than the third stage (which runs pplacer), is very fast and uses little memory.

Table 1 compares pplacer-SCAMPP (i.e. pplacer used within the SCAMPP framework) with APPLES and EPA-ng with respect to delta-error (a measure for the increase in topological error in the tree produced by the phylogenetic placement method; see [81,86] for the definition). The placement methods are given full-length sequences in the true alignment and place these sequences in a leave-one-out strategy into the model tree on the remaining sequences, with trees varying from 5000 to 200 000 sequences. EPA-ng fails to be able to place into the largest trees due to memory requirements, but APPLES and pplacer-SCAMPP succeed on all trees. Note that pplacer-SCAMPP has the lowest placement error of all methods.

**Table 1.** Average delta error (Δ*e*) for phylogenetic placement methods in backbone trees of size *n*. Analyses were limited to 64 Gb of memory.

	*n* = 5000	*n* = 10 000	*n* = 50 000	*n* = 100 000	*n* = 200 000
			Δ*e*		
pplacer-SCAMPP	0.150	0.132	0.085	0.084	0.075
EPA-ng	0.239	0.219	X	X	X
APPLES	0.366	0.330	0.239	0.247	0.250

### (b) Adding species to species trees

While the methods above focused on adding sequences into gene trees, adding species (represented by genome-scale data) into species trees is another kind of phylogenetic placement problem. One such method is MGPlacer [88], which uses reads from across a genome to place a genome into a species tree. Other approaches, such as INSTRAL [89], have been developed that consider heterogeneity across the genome due to processes such as ILS. Given an existing species tree *T*, INSTRAL will add the new species into the existing tree to optimize the quartet tree support for the extended species tree (i.e. INSTRAL extends the theoretical approach in ASTRAL). Another new method is DEPP [90], which computes distances using a deep neural network and then runs APPLES to place the new species into the tree. Of these methods, MGPlacer has the desirable property in that it can decide to not add a sequence into a tree due to insufficient evidence of homology.

## 6. Concluding remarks

This review has described some of the significant innovations over the past few years in the development of methods for multiple sequence alignment and phylogenetic tree estimation that provide high accuracy on very large datasets (even up to 1 000 000 sequences). Because so many of the methods discussed in this review are extremely new, additional studies are needed to explore and understand the conditions under which these methods are reliably more accurate than alternative methods, and our review has suggested some potential directions where such study is needed.

Owing to space constraints, we did not discuss all the relevant problems for large-scale tree estimation, including how to efficiently and accurately estimate numeric parameters (e.g. branch lengths) or evaluate branch support in a large tree. There is active work on these problems (e.g. [6,91,92]), but each of these problems is likely to remain an important direction for research. We also did not address Bayesian inference, which is an important class of phylogenetic methods [78,93,94]. Bayesian methods, such as MrBayes [95], are well established in the research community and have been shown to provide highly accurate point estimates of alignments, gene trees and species trees; however, most Bayesian methods use MCMC (Markov Chain Monte Carlo) and are computationally intensive on large datasets since convergence to the stationary distribution is required for high confidence in an accurate result. Some progress has been made on improving the scalability of these point estimations using Bayesian methods, e.g. by using divide-and-conquer to break a large dataset into subsets or constraining the search space (e.g. [20,58,96,97]). However, Bayesian methods produce distributions from which point estimates can be obtained, and these distributions have significant additional value since they enable uncertainty quantification. Scaling Bayesian methods to large datasets so that a good estimate of the distribution can be obtained is of great interest, but is generally not enabled through the techniques that focus on scaling the point estimates. Here we note that [43] has made some progress in scaling MrBayes, suggesting that additional effort in this direction is merited. In general, fully scaling Bayesian methods requires additional techniques beyond those explored in this survey.

We also did not discuss in full how different causes for gene tree discord can affect species tree estimation. As we have seen, even in the presence of ILS and GDL, a tree is a reasonable model for the evolutionary relationships between the species. However, some biological processes, such as gene flow, horizontal gene transfer and species hybridization, may require graphical models of evolution called ‘explicit phylogenetic networks’ [98–100] that are not purely tree-like. For example, in a hybridization network, a hybrid species will have two parents rather than one, while in a network representing evolutionary relationships that include HGT events, there will be two types of edges: those depicting vertical transmission and those depicting HGT events.

Under some conditions, such as with limited gene flow or relatively small amounts of random HGT, the estimation of the ‘main’ tree within a phylogenetic network is a reasonable objective [101–104], especially if a well-established subset of the genes are believed to evolve down this main tree [105].

A simulation study in [101] evaluating methods for estimating the main tree in the presence of gene flow showed that using PhyloNet [106] to construct a hybridization network (under ML) and then suppressing the ‘minor’ hybrid edge produced the most accurate results, followed by ASTRAL, NJst [107] and finally concatenation. Thus, ASTRAL provided superior accuracy compared to the other tree inference methods, but a phylogenetic network approach was key to obtaining high accuracy. Because of this performance, Solís-Lemus *et al*. [101] argue for the use of likelihood-based phylogenetic network methods for estimating the ‘main tree’ in the presence of gene flow. Unfortunately, likelihood-based methods for estimating explicit phylogenetic networks are enormously computationally intensive and even the most scalable such methods are limited to a few tens of species [108–111].

Therefore, method development for explicit phylogenetic network estimation is also needed. Alternative approaches, such as providing approximate representations of evolutionary relationships (e.g. clusterings and visualizations) rather than trees or explicit phylogenetic networks, are also valuable, especially when evolutionary relationships are complex and the dataset is very large; the paper by Lees *et al.* [112] is a promising example of such an approach.

This study did not discuss all the recent advances in large-scale alignment and tree estimation, and some of these may provide even better scalability and accuracy. For example, there are new methods for large-scale ML tree estimation (e.g. Very Fast Tree [113]), new techniques to speed up co-estimation of gene trees and species trees [96,114], and even divide-and-conquer approaches to phylogenetic network estimation [115]. This continued effort to develop methods that are highly accurate and scalable leads us to the optimistic prediction that the next 5–10 years will result in new scalable methods to estimate accurate alignments, trees and even phylogenetic networks, and that these methods will enable biologists to make discoveries on the large and ultra-large phylogenomic datasets that they assemble.

## Data Availability

This article has no additional data.
